# Scale-Up Technologies for the Manufacture of Adherent Cells

**DOI:** 10.3389/fnut.2020.575146

**Published:** 2020-11-04

**Authors:** Caroline Faria Bellani, Jila Ajeian, Laura Duffy, Martina Miotto, Leo Groenewegen, Che J. Connon

**Affiliations:** ^1^International Center for Life, Biosciences Institute, Newcastle University, Newcastle upon Tyne, United Kingdom; ^2^CellulaREvolution Ltd, International Center for Life, Newcastle upon Tyne, United Kingdom

**Keywords:** cultured meat, scale-up cells manufacture, bioprocessing, continuous bioprocessing, adherent cell manufacture, bioreactors

## Abstract

Great importance is being given to the impact our food supply chain and consumers' food habits are having on the environment, human health, and animal welfare. One of the latest developments aiming at positively changing the food ecosystem is represented by cultured meat. This form of cellular agriculture has the objective to generate slaughter-free meat products starting from the cultivation of few cells harvested from the animal tissue of interest. As a consequence, a large number of cells has to be generated at a reasonable cost. Just to give an idea of the scale, there were billions of cells just in a bite of the first cultured-meat burger. Thus, one of the major challenges faced by the scientists involved in this new ambitious and fascinating field, is how to efficiently scale-up cell manufacture. Considering the great potential presented by cultured meat, audiences from different backgrounds are very interested in this topic and eager to be informed of the challenges and possible solutions in this area. In light of this, we will provide an overview of the main existing bioprocessing technologies used to scale-up adherent cells at a small and large scale. Thus, giving a brief technical description of these bioprocesses, with the main associated advantages and disadvantages. Moreover, we will introduce an alternative solution we believe has the potential to revolutionize the way adherent cells are grown, helping cultured meat become a reality.

## Introduction

In the last 20 years there have been considerable advances in disciplines such as biology and biotechnology each generating important breakthroughs in tissue engineering and regenerative medicine. As a result, considerable progress has been made in different fields leading to the development of multiple cell-based therapies, new and more effective biologics as well as improved approaches to regenerate damaged tissues. Moreover, this state-of-the-art knowledge fostered the development of new fields such as cultured meat (1, 2). Indeed, this form of alternative protein production relies upon applying and manipulating cutting edge technologies in cell culture, tissue engineering and bioprocessing to achieve the *in vitro* production of slaughter-free meat. In addition, this new but rapidly developing field demands a strong interdisciplinary effort spanning from molecular and cell biology to engineering.

Scientists working in the field of cultured meat are facing numerous challenges, largely the scale and type of problem depends upon the approach they are taking to generate their final products–lab grown meat (3, 4). One of the most critical decisions each manufacturer must make is which scale-up bioprocessing approach they should take. As in other fields such as allogeneic cell therapy, there is the necessity to efficiently generate large numbers of cells (5, 6). For instance, production of cultured meat will require the producers to culture billions of cells (10^12^-10^13^ cells to generate ~10–100 kg of meat) while aiming at using limited space, time, and resources to keep the costs down (7). To give a general idea of the scale, to satisfy only 10% of the world meat consumption (~30 × 10^6^ t/y), we would need at least 2 × 10^6^ m^3^ bioreactor volume (corresponding to ~200,000 × 100 m^3^ bioreactors). Growing this number of cells is extremely challenging since scalability for adherent cells has never being proven at such high scale.

Thus, choosing the right scale-up process is essential not only to meet the required cell demand, but also to limit the costs of manufacturing. As an example, when Professor Mark Post took on the exceptional challenge and created the first “cultured burger,” adherent cells were grown upon a surface made of thousands of layers of tissue culture plastic stacked on top of each other, ramping production costs to around €250,000 for that single burger (1). Indeed, this culture system has significant limitations in terms of scalability (currently limited to the production up to 10^11^ cells), with unfavorably low surface to volume ratio, as well as lacking control over pH, gas, and metabolite concentrations (8).

**Figure 1:**
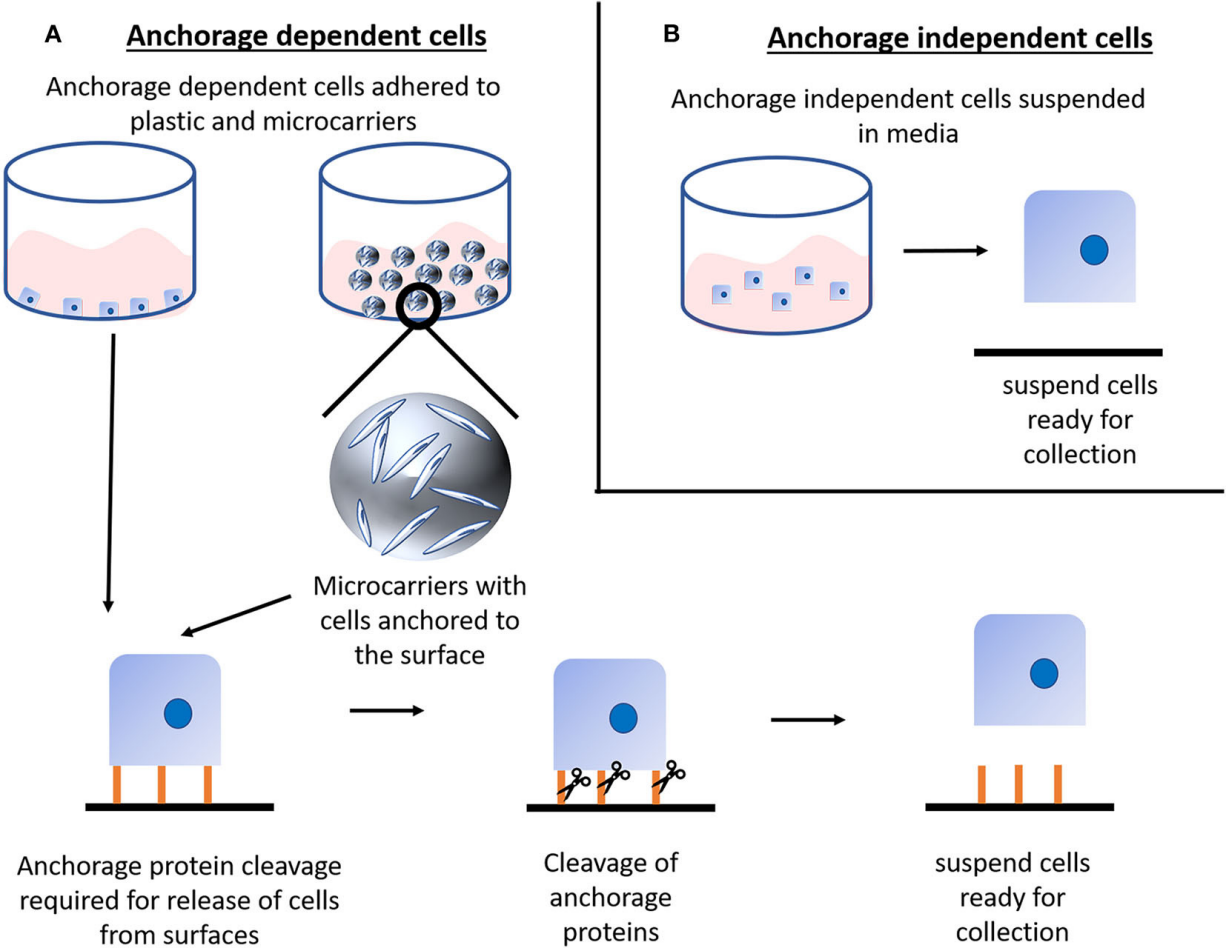
Cell harvesting: **(A)** Anchorage dependent (adherent) cells require adherence to a surface for sustained and healthy culture, this anchorage can be to either common tissue culture plastics or to microcarriers. Although microcarriers themselves are in suspension it is important to note that the cells are still anchored to the microcarrier and thus require the same cleavage of anchorage proteins as cells anchored to tissue culture plastics. Classically, anchorage proteins are cleaved either via enzymatic or mechanical means. Such cleavage releases the cells from the surface and into suspension for collection. Anchorage dependent cells typically cannot survive long in suspension conditions, hence the requirement for batch cleavage. **(B)** Anchorage independent (suspension) cells do not require surface adherence to be viable and proliferate, thus they are readily available for collection and easily adaptable to bioprocessing.

A major scale-up challenge is for those cells that are anchorage-dependent, commonly referred to adherent cells. These are the most common form of animal cell and are widely used in all fields (i.e., regenerative medicine, cell therapy, to produce biologics etc.), including the production of cultured meat (mesenchymal stem cells, muscle satellite cells, and induced pluripotent stem cells are just some examples) (1, 9). These cells need to adhere to a surface in order to remain viable and proliferate. Thus, for an efficient *in vitro* cell expansion system, there is an urgent need for improved bioprocesses which enable a more favorable surface to volume ratio, tighter control over critical growth parameters, better optimized dissociation from the growth surface and more efficient final cell harvest. In order to improve on the surface to volume ratio, two strategies are employed typically: (i) adapt the cells to grow as anchorage-independent (suspension) cells or (ii) use suspension culture systems (such as microcarriers) where cells are attached to and proliferate upon carriers that are constantly agitated to remain in suspension (Figure 1). Adapting adherent cells to grow as suspension cells is often laborious as it can take months to achieve and ultimately can often be unsuccessful as not all cells are capable of fully adjusting to this new growth condition (10). Moreover, if the adaptation step is successful, it remains important to closely monitor the system and regularly dissociate cell aggregates to prevent spontaneous differentiation and the formation of necrotic cores within the aggregates. On the other hand, more common is the use of suspension culture systems like microcarriers since they can be used in different bioprocesses and offer an adhesive surface whilst their mass is small enough to be suspended in the cell culture media under stirring (Figure 1A).

We are aware that there might be studies and strategies exploring the production of cultured meat using cells adapted to grow in suspension. However, bioprocesses to scale-up suspension cells are less challenging than for adherent cells as the need for specialized growth surfaces for the cells to adhere to is removed. Moreover, the footprint and the complexity of the cell collection step are reduced and are well-established within the industry (Figure 1B).

In light of this, within this review paper we have decided to focus on the manufacture of adherent cells highlighting existing and future technologies to their scale-up.

## Key Parameters and Considerations on Scale-Up

Before starting to list and technically describe all the different scale-up technologies, it is important to highlight what key parameters need to be considered when designing a bioprocess that aims to successfully manufacture a large number of adherent cells.

### Availability of Key Elements

Oxygen, carbon dioxide and nutrients need to be added to the media in order to support cell growth within the expansion system (11). Oxygen can be added either in the form of aeration through sparges within the bioreactor, or upstream to ensure the media is saturated with dissolved oxygen. Bubble aeration through sparging is traditionally used to supply oxygen in large scale bioreactors, however alternative bubble-free aeration methods exist such as use of gas permeable silicone tubing for feed piping, or an external media aeration device. When the oxygen falls below the cell metabolism requirement level, the speed of respiration slows down, negatively impacting cell growth and consequently product quality (12).

Depending on the buffer used to maintain pH 7.2–7.4 within the bioreactor, provision and maintenance of carbon dioxide concentration may be required. In large-scale culture systems, high concentration of carbon dioxide (CO_2_) is often considered undesirable (13). When CO_2_ is above a certain level, cell growth can be inhibited, and the product quality compromised since cell-derived polysaccharides (N-glycans) can be affected due to disruption of the intracellular pH environment (14, 15). In light of this, sensors to monitor and feedback control systems on these key elements are critical.

Regarding the availability of nutrients, the most common strategy to feed the culture medium into the process is the fed-batch system. Fed-batch is an operational technique used in a variety of biotechnological processes where one or more nutrients are fed (supplied) to the bioreactor during the culture period and in which the product(s) remain in the bioreactor until the end of the run (16, 17). The culture medium is typically added through perfusion leading to less variation in nutrients and better cell yields (15). Perfusion of the culture medium allows for monitoring and control of the process conditions, which, as mentioned previously, is critical in the development of a reproducible manufacturing process.

### Shear Stress

On one hand, the dynamic culture in bioreactors enhances nutrients transport and waste removal, but on the other it is exposing the cells to increased fluid shear stresses (18, 19). Cells that are grown under these conditions respond to these external *stimuli* in different ways, depending on the cell type (19). Considering that bioreactors aim at recreating an *in vitro* environment that is very similar to the *in vivo* condition, shear stresses can be modulated *ad hoc* depending on the cell type and on the application (15). For instance, osteoblasts and mesenchymal stem cells (MSCs) have been shown to directly respond to shear stress (20). Indeed, mechanical stimulation through fluid shear stresses seems to promote bone differentiation and mineralization (21). However, there are cases when these forces impact negatively cell viability, growth, and cell behavior (22, 23). In this regard, the pharmaceutical and cell therapy industries have raised concerns and are still looking to minimize and optimize the stirring method to reduce the impact of fluid shear stress on the cells within the bioreactor.

### Footprint

Regarding the equipment involved in scale-up processes, it is important to consider the physical space occupied by a certain machine, aka its footprint. The size, type and number of bioreactors will have an impact on the environment, overall costs, energy consumption, resources, handling, product quality, and reproducibility (24, 25). Large footprints are generally more associated with the expansion of adherent cells, since they are required to adhere to a substrate (26). Currently, the most common technologies aiming at reducing the footprint during the expansion of adherent cells are based on cultures using microcarriers-based and hollow-fiber bioreactors (that will be discussed in the following chapters).

### Traditional and Intensified Processes

**Figure 2:**
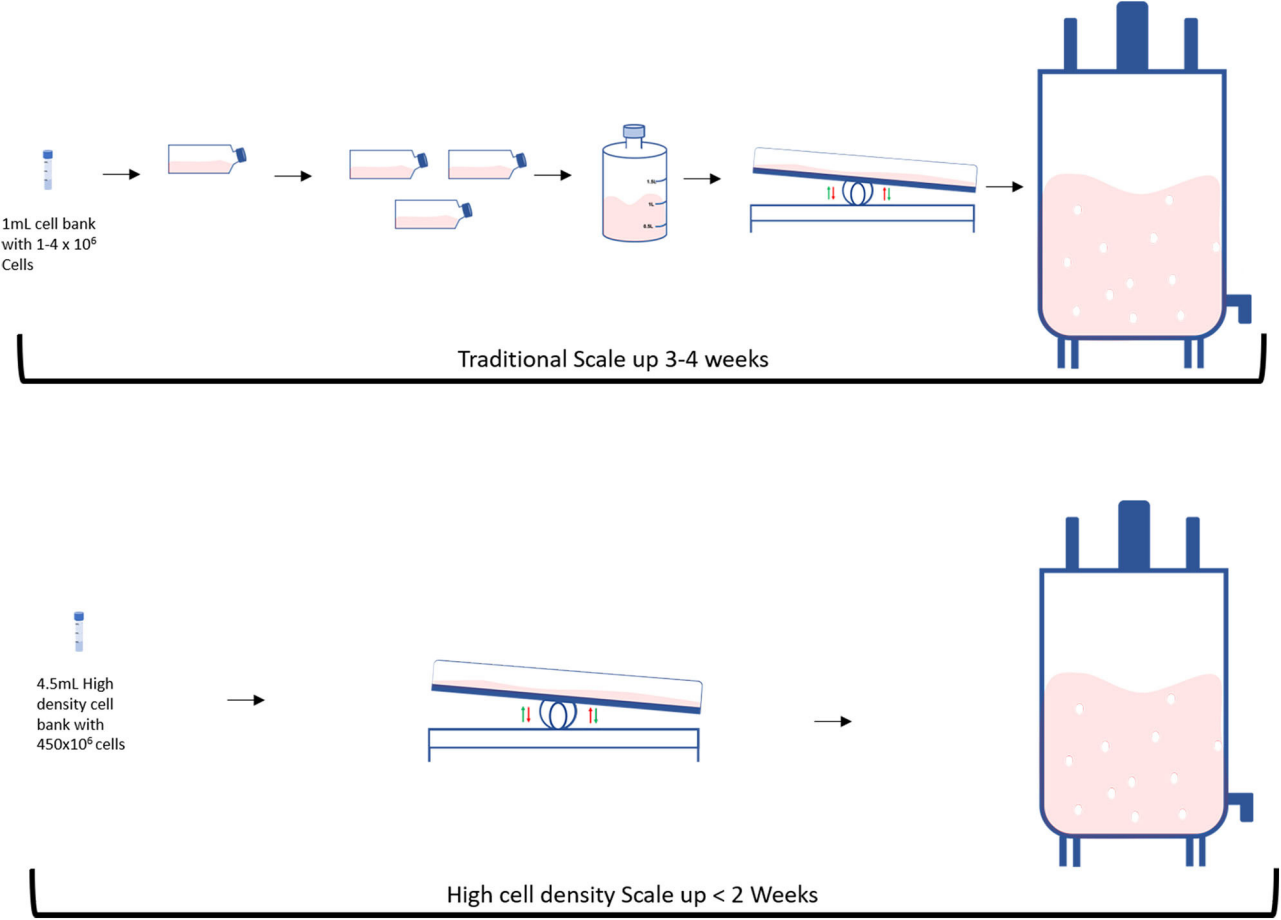
Differences between traditional and High intensity scale up: **(Top)** Traditional and **(Bottom)** High intensity methods.

**Figure 3:**
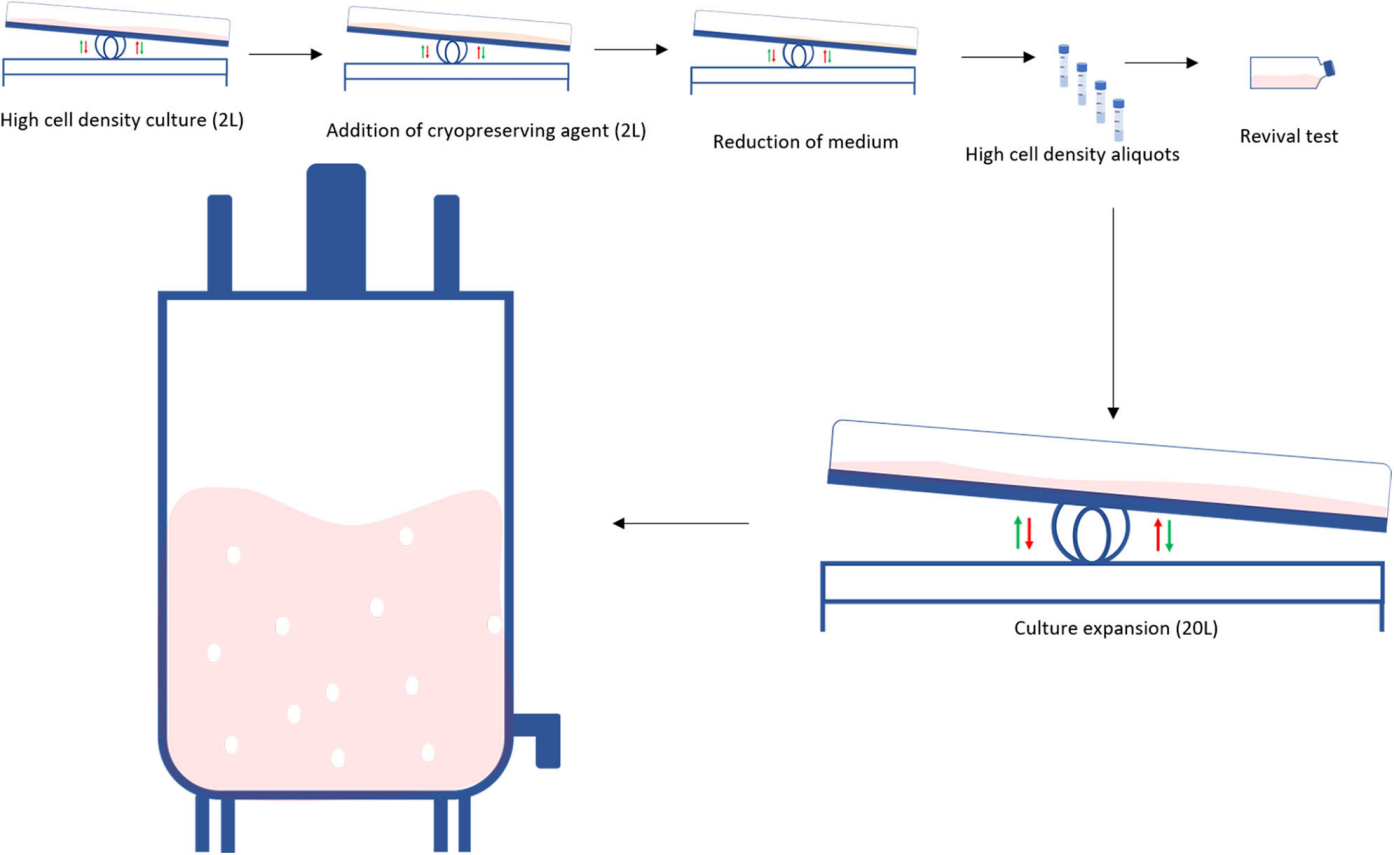
Obtaining a high-density cell bank: High density cell banks are used to reduce the required steps in the traditional scale-up process to generate larger numbers of cells. Initially, cells are grown at a high cell density in a perfusion bioreactor, a cryopreservation is added to the culture and the volume is reduced. Cells are then banked as high-density aliquots in cryovials. When required, a test vial is revived. If the revival is successful, the high-density aliquots are seeded into a perfusion bioreactor which is subsequently seeded into a larger bioreactor.

A traditional bioprocess consists of expanding an initial cell aliquot starting from a small vessel and then progressively increasing the vessels' size every time the cells reach confluency (Figure 2 top). This process can take 3–4 weeks and requires frequent and multiple manual operations to generate a sufficient cell number to progress to the next stage. Tao et al. proposed an alternative system to both speed-up the time and reduce the number of steps during the scale-up process by producing high density (HD) cell banks (27) (Figure 2 bottom). Traditional vials contain 1–4 million cells, whilst each of these cell banks generally contain 450 million cells. Such HD cell bank vials are then used to inoculate several rocking motions (wave movement) bioreactors, eliminating several intermediate expansion steps in shake flasks (Figure 3). In this way, the manipulations in the laminar flow hood are significantly reduced, decreasing the associated labor and the potential risk of contaminations. This strategy is capable of reducing process time up to 9 days and improves operational success in seeding expansion steps.

### Scaling-Up and Scaling-Out

**Figure 4:**
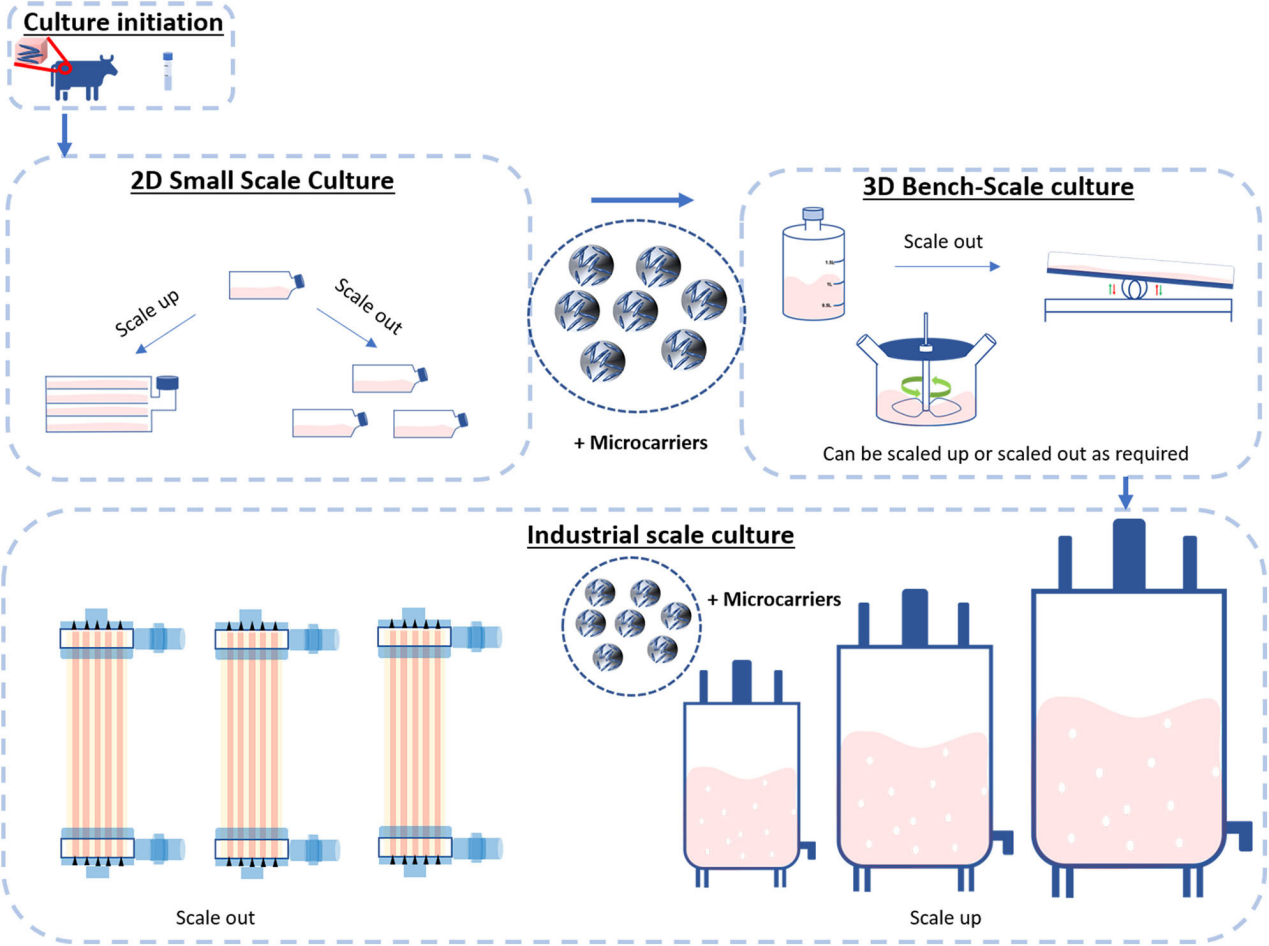
Scale-up and Scale-out. Cultures are typically initiated from cryopreserved stocks or biopsies and cultivated in small-scale cultures such as flasks. These flasks can be scaled up with the use of microcarriers or scaled out with the use of hyper or multilayered flasks. The process then enters the bench scale stage. Here, the use of larger vessels such as roller bottles, perfusion bioreactors and spinner flasks can be deployed to both scale-up and scale-out the process. When required, the process then enters the industrial scale. This stage of bioprocessing enters two distinct streams: bioreactors scale-out (multiple bioreactors of the same size) or scale-up (further processing up to a single bioreactor).

In the biotechnological and bioprocessing industries, scale-up and scaling-out are two widely employed strategies to generate large numbers of cells. Scale-up systems progressively increase the surface area/culture volume as the cell number raises (28). Scale-out systems are based on the use of multiple culture vessels/bioreactors working in parallel (Figure 4). There are advantages and disadvantages associated with each approach. For instance, compared to scale-up processes, scaling-out can better deal with changes in product demand and improves process performance, however reproducibility can be difficult to achieve. Instead, scaling-up processes are more difficult to handle and control due to the high working volumes involved, but it can lower the costs of goods in the long term (28, 29).

### Monitoring Systems

Bioreactor monitoring systems can be divided into three types: “offline,” “at line,” and “online.” Offline monitoring can be defined as a manual operation consisting in removing a sample from a bioreactor and processing it in the laboratory. The at line system differs from the offline in that the sample, despite being removed from the bioreactor, is being tested right next to it. However, an online system provides the opportunity to test samples both *in situ* and *ex situ*. In the *in situ* system, an in line analyser tests the sample and then returns it back into the bioreactor; while in the *ex situ* approach the sample does not return to the bio-analyser after been measured (30). While in line analytical methods to monitor the pH, dissolved oxygen and temperature are already available, other parameters like the substrate density are still being measured offline through laborious and error prone methods (31). An example in which components of a bioreactor can be monitored offline, with the help of biomass separation methods, is the High-Performance Liquid Chromatography (HPLC) system. The advantage of using HPLC is that components of the media will be separated by adsorption, liquid-liquid interaction, or affinity separation. The downside of using manual sampling methods is time consumption as well as not being able to test the samples in real time (32). Monitoring a bioreactor in real time is of major importance as it can lead to higher efficiency, productivity, product quality and overall cost reduction (33). For instance, cell density and viability are two of the most critical factors for a bioprocess and they should be measured in real time. Instruments facilitating these measurements are based on optical density, fluorescence or conductivity and are providing online measurements which subsequently will be verified using offline methods such as microscopy (34). At-line monitoring of substrate and reagents density can be performed using optical sensors, ultrasound sensors, UV-Vis, fluorescence and RAMAN spectroscopy (31). RAMAN and near infra-red (NIR) spectroscopic methods are popular in the pharmaceutical industry and are based on the interaction between light and matter. Both RAMAN and NIR are non-invasive methods that can provide useful information about cell culture bioprocesses albeit the interpretation of the spectra is complex and needs chemotactic and multivariate method (35). Near Infra-Red spectroscopy (NIR) is a popular method for in line bioprocesses measurements and combined with multivariate data analysis provides the opportunity to perform a real time measurement of a number of parameters (36). The information regarding the spectra is obtained using an FTIR spectroscope, acquiring the data with a probe that is inserted into the bioreactor system (37). NIR advantages include easy maintenance, being non-invasive and the identification of multiple analytes in the media. However, FTIR probes are expensive and the immersion of probes into the bioreactor broth requires thorough sterilization (31). Finally, it is worth mentioning *in situ* microscopy, this is capable of taking images of cells from inside a bioreactor without the need to take the sample out (38) since the field of view is fixed (34). Overall, despite the increasing scale of bioreactors, traditional monitoring methods are still in use, suggesting the need in implementing more reliable, automated and real-time systems (35).

## Small Scale Technologies–or Compact Technologies

In this section we will describe four of the most commonly used devices to scale-up adherent cells at bench scale. An overview of the main technical characteristics and relative advantages/disadvantages is also presented in Table 1.

**Table 1:** Table summarizing the advantages and limitations of the described small-scale systems.

**Device**	**Surface area (cm^**2**^)**	**Cell densities (ml^**−1**^)**	**Features**	**Disadvantages**
T-Flask	25–225	1 × 10^5^	• Low cost • Easy to use • Easy cell adaption • No special extra equipment required	• Scaling-out can be labor intensive • Inconsistency
Multi-layer flask	525–18,000	1 × 10^5^	• Increased surface area compared to T-Flask • All layers are passaged at once	• Increasing in layers numbers impact in the difficult to handle it • Heterogeneity within different layers
Roller bottle	850–1,700	1 × 10^5^	• Rotation provides better distribution of nutrients and oxygen • Require less media compared to planar flasks	• Scaling-out can be labor intensive • Require extra setup to roll bottles.
Spinner flasks associated with microcarriers	380/g	1.7 × 10^6^	• Improved surface and cell yield • Homogeneous mass transfer • Relatively low-cost • Optimization prior to industrial scale-up in suspension	• Shear stress can be harmful to cells • Require optimization with cell line and microcarriers

### T-Flasks

**Figure 5:**
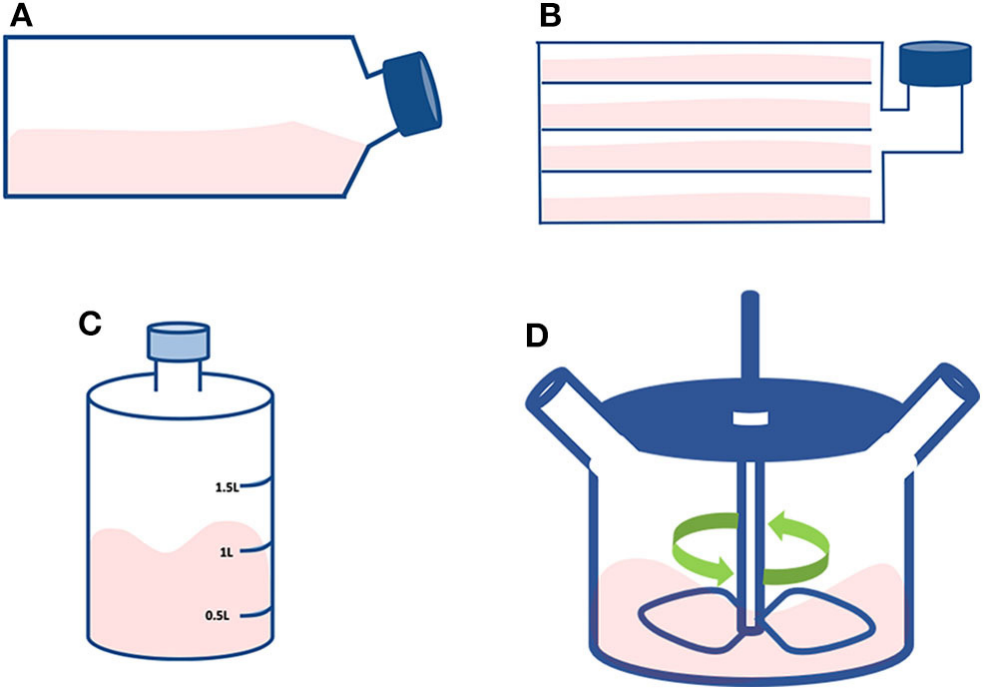
Small scale technologies. Schematic representing the discussed compact technologies: **(A)** T-flask; **(B)** Multi-layered flask; **(C)** Roller bottle; and **(D)** Spinner flask.

T-flasks are the most commonly used plastic consumables for early stage cell expansion, usually when growing cells starting from a cryovial (Figure 5A). T-flasks vary in size, ranging from a culture area of 12.5–225 cm^2^ and are made of disposable plasma treated polystyrene, or tissue culture plastic (TCP) (39, 40). While conveniently economical, these flasks are labor-intensive and become cost-inefficient when expanding cells beyond bench scale, mainly because of their high footprint.

### Multi-Layered Flasks

They are large T-flasks composed of stacked flat surfaces (Figure 5B). The aim is to increase the available surface by incorporating a multi-tray unit reaching a total area that depends on the number of layers, but generally reaching up to 2.5 m^2^ (39, 41). This type of flask must be treated as an individual unit with the cells from each layer to be seeded, cultured and detached at the same time. Although being a useful device for scaling-up at bench scale, there are concerns regarding the cell quality and the associated labor intensity. For instance, there might be a heterogeneous availability and distribution of nutrients and gasses between the different layers of the flask (41). Moreover, simple operations like cell seeding, media change and cell detachment/harvest become challenging due to their size and weight. In this respect, system automation would greatly improve these day-to-day operations.

### Roller Bottles

These bottles consist of cylindrical vessels to grow cells in a dynamic system (Figure 5C). They are usually placed in a heated environment on a rack that slowly revolves (ranging between 5 and 240 revolutions/hour). They are inexpensive and are a common method used for the initial scale-up of adherent cells (42). The cells attach and cover the inner surface of the bottle; hence the cells are cyclically bathing in culture medium and exposed to gases. In addition, the rotation provides a level of mixing, preventing gradients from forming within the medium that may affect cell growth. In this system, the cells are most of the time covered by a thin layer of medium, thus facilitating superior gas exchange (18). The surface available for cell expansion is between 500 and 1,700 cm^2^, in a total volume ranging 1 to 1.5 L, suitable for culture volumes of 0.1–0.3 L (39). Like static flasks, rotating flasks are also labor intensive. For high cell numbers, a further constraint of a roller bottle process through scaling-out is the limitation in the control of O_2_ and CO_2_ in both the gas and the liquid phase of culture (39, 42).

### Spinner Flasks

These devices are flat-bottom flasks commonly used at a bench-scale for stirred suspension cultures that can be used to initially validate microcarriers and media composition (43) (Figure 5D). The culture is maintained in suspension and the stirring is achieved by a magnetic stir bar, also called magnetic driven impeller (44). The media is inoculated with cells to fill the flask with a volume of 100–200 ml at a stirring speed of 50 rpm (45). Compared to the solutions mentioned earlier, spinner flasks can generate high cell numbers, provide a better aeration system, a more homogeneous nutrient supply, longer culture period and reduced costs. Microcarriers can be added to spinner flasks mainly to do preliminary tests before moving to larger bioreactors (7). Microcarriers are small spheres with a diameter ranging between 90 and 300 μm and available in different sizes, materials, coatings, and surface charges (46–48). Different sizes and materials impact on the microcarriers seeding density and cell harvesting methods (48). Cell adhesion treatments can enhance cell attachment and promote cell spreading (49). As the choice of the microcarrier depends on the cell type, product, and operational set-up, it is highly recommended to run preliminary tests with different microcarriers (48, 50, 51).

The critical following steps are: (i) cell dissociation from the carriers and (ii) harvesting cells from the media (7, 49). Many studies have reported challenges in efficiently detaching the cells from the carriers using classic enzymatic methods (7, 49, 52). To mitigate this problem, current solutions include coatings with thermo-responsive polymers (e.g., pNIPAAm) (53), degradable (e.g., made of PGA) (54) and edible (e.g., made of alginate or chitosan) microcarriers (7).

## Large-Scale Bioreactors–or Industrial Scale

In this Chapter we will describe four of the most commonly used devices to scale-up adherent cells at industrial scale. An overview of the main technical characteristics and relative advantages/disadvantages is also presented in Table 2.

**Table 2:** Table summarizing the advantages and limitations of the described large-scale systems.

**Device**	**Max Capacity^**a**^**	**Cell density (ml^**−1**^)**	**Features**	**Disadvantages**
Wave bioreactor (associated with microcarriers)	20 L/0.02 m^3^	2 × 10^6^	• Tool for intensified scaling-up • Low shear stress • Operation in different batch modes	• Scale-up to >100 L is challenging • Large space is needed
Stirred tank (associated with microcarriers)	2,000 L/2 m^3^	2 × 10^6^	• Easy of scaling up from benchtop to factory • Bioprocessing is well-understood • Flexible and automatic platform for very high-volume bioprocess	• Require optimization with cell line and microcarriers • Large volumes required • High shear stress
Packed bed	500 m^2^/ 0.03m^3^	3 × 10^6^	• High density cell culture due to large surface available • Operation in different batch modes • Cell passage less frequent	• Packing material difficult cell harvest • Concentration of gradients
Hollow fiber bioreactor^b^	150 cm^2^/ml^−1^ 0.00007 m^3^	1 × 10^9^	• Increased surface to volume ratio • *In vivo*-like tissue structure (blood vessels)	• Difficult to harvest cells • Concentration of gradients

a *Commercially available*.

b *Variable maximum capacity, as various cartridges can be connected in parallel*.

### Rocking Motion Bioreactors

**Figure 6:**
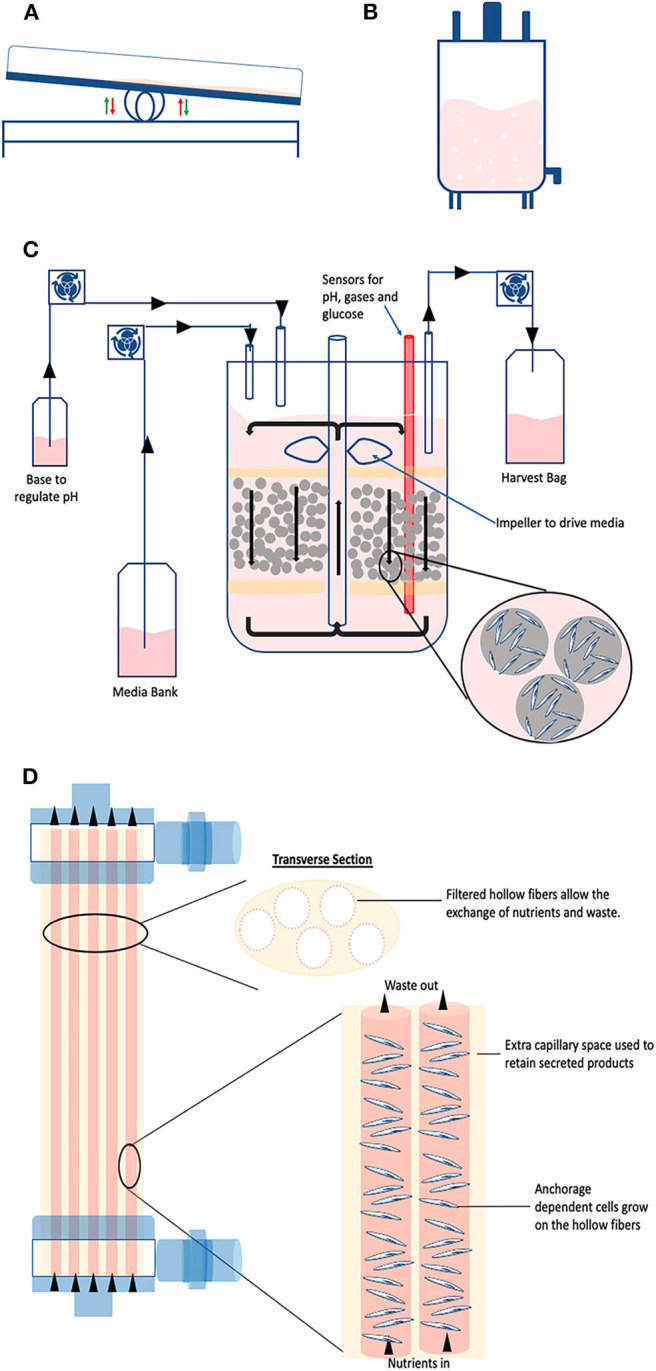
Large scale technologies. Schematic representing the discussed industrial technologies: **(A)** Wave; **(B)** Stirred tank; **(C)** Packed bed; and **(D)** Hollow fiber bioreactors.

This type of reactor utilizes the wave motion of culture medium generated by a rocking platform to provide a cell-beads (microcarrier) suspension (Figure 6A). The beads are placed inside a disposable bag with ports allowing for air circulation and bag inflation (55). The disposable bag system has advantages for clinical applications in terms of safety providing the ultimate ease in operation and protection against cross-contaminations (55, 56). The chamber is placed on a special rocking platform causing low/negligible shear stress to the cells (55, 57). The agitation provides proper mixing and mass transfer while the circulating air provides the necessary oxygen exchange (57). Of note, new rocking motion bioreactors models have a higher mass transfer than the standard wave type bioreactors while inducting a relatively low shear stress. It is possible to connect culture medium bags for perfusion via additional ports and it can operate via batch, fed-batch, repeated fed-batch, and perfusion mode (12). This setting facilitates scale-up and automation, which has been demonstrated for culture volumes up to 500 L (58). Such a system is widely used for the expansion of mammalian cells, for example embryonic feline lung fibroblasts (59), neutrophils from HSCs (58), and T cells (60). Considering that these reactors allow high cell yields, they are the platform of choice when expanding High Density cell banks obtained from intensified processes (27).

### Stirred Tank Bioreactors

Giving the existing broad knowledge on stirred systems, stirred tank reactors are possibly the most used system for large-scale culture of mammalian cells (15, 61) (Figure 6B). They apply the same operational principles as the spinner flask (agitation in a tank via an impeller), just in much larger volumes which can reach up to 2,000 L in a single vessel (62). The impeller keeps the solution in agitation to maintain the particles (i.e., organoids, suspension cells, or microcarriers) in suspension whilst homogenizing the distribution of oxygen, nutrients, and heat (63). The tank provides a closed and automated platform and can operate in different modes, such as batch, fed-batch, and perfusion (15). Considering that it is a suspension culture system, it offers the typical advantages of optimized footprint. When it is used to grow cells attached to microcarriers, it can provide *in situ* assistance in dissociating the cells from the carriers when cells reach confluency (18, 52). The strategy is based on coupling the addition of trypsin with intense agitation. The generated shear stress improves the cell detachment efficiency, thus increasing the final yield. In this particular case, fluid dynamics tells us that this brief and intense shear stress does not damage the cells because the detached cells are smaller than the Kolmogorov scale of turbulence (52). Industrial stirred tank bioreactors are available as single-use, however they are traditionally made of stainless steel (cGMP material) considering it is easy to clean, well-compatible with biologics and highly resistant to pressure and erosion (11).

### Packed Bed Bioreactors

Also called fixed bed, they consist of a hollow tube packed at the bottom with immobilized surfaces such as scaffolds, microcarriers or porous fibers (Figure 6C). The cells are seeded on the fixed bed while fresh media is continuously circulating within the system transferring oxygen and supplying nutrients, whilst providing a large surface to volume ratio for cell attachment and expansion (18, 64). This impacts cell passaging: due to the high surface area, cells can be passaged less often, thereby there are savings on costs of culture media and operations (57). They are commercially available at cGMP bench scale (up to 4 m^2^) and for industrial scale manufacturing (up to 500 m^2^) (18, 57). High cell densities of 5.1 × 10^8^ cells/mL have been reported with packed bed bioreactors (64). Very early progenitor cells (CFU-GEMM) were expanded up to 4.2-fold while later progenitor cells (CFU-GM and BFU-E) exhibited up to seven-fold and 1.8-fold expansion, respectively (65). Moreover, an average seven-fold expansion of MSC was reported with a starting cell density of 6.0 × 10^7^ cells, after 7 days of culture (66). Additionally, the perfusion operation offers the monitoring and control of the process conditions (18). It has to be noted that the structure of the reactor does introduce a risk of formation of an axial and radial concentration of gradients, especially at a large-scale (18). Cell harvesting can also be problematic due to the presence of high cell densities and the difficulty of effectively introducing the detachment supplements into the culture (18).

### Hollow Fiber Bioreactors

They consist of a cylindrical chamber stacked with semi-permeable hollow fibers (Figure 6D). Cells can be inoculated both within the fibers and on the extracapillary surfaces, permitting high cell densities in the order 1.0 × 10^9^ cells/mL (67, 68). The fibers mimic blood vessels in coordinating nutrient supply and removal of waste while oxygen exchange is managed by diffusion between intra-capillary and extra-capillary spaces (57, 67). The culture medium can flow through the fiber or chamber or both using proper channels and ports. Depending on the inoculation method, pore size for the semi-permeable membrane can be chosen to determine which particles shall pass through or retained by the membrane. For instance, if the cells are inoculated in the intracapillary surface, then the media is perfused from outside or extra-capillary space. This flow operation is known as intra-capillary inoculation with extra-capillary perfusion (69). Due to its perfusion nature, it allows automated monitoring and control of metabolites concentration which is important in maintaining process consistency (18). However, there is the potential dissociation of longitudinal concentration gradients as culture medium or dissociation reagent flows down the fibers, meaning the nutrients distribution can be inconsistent along the hollow fibers (67). Strategies to overcome these limitations include the use of oxygen carriers that increase the flow rates and/or rotate the hollow-fiber bioreactor in timed cycles to reduce oxygen gradients (70). Hollow fiber bioreactors have been employed to expand MSCs and human umbilical cord derived HSCs. The culture was carried out with seeding densities of 800,000 cells/ml to demonstrate a semi-continuous production model with up to 14,288-fold expansion while maintaining pluripotency markers (69, 71). In order to increase cell production, it is also possible to connect various units in parallel (scale-out) (72).

## Continuous Cell Culture as an Alternative Approach

More than 30 years ago, the concept of continuous bioprocessing was introduced as an alternative to batch production; in general terms it means creating a continuous process of turning flowing raw material into intermediate or final products (73). A broad range of industries have adopted continuous processes, spanning chemical and oil refineries to food and life sciences (74–76). The reason for this implementation relates to the proven benefits continuous processing brings to reducing process cycle times, materials and energy used as well as waste production (77). Recently, the biopharmaceutical industry, with the rise of cell based therapies, had to become more competitive in terms of bioprocessing to reduce the costs of manufacturing (78). For this reason, great efforts have been made in implementing continuous platforms to achieve better efficiency and become more cost effective (79). Focusing on this sector, a more efficient *in vitro* cells expansion at large scale is still demanded for adherent cells.

The way of culturing adherent cells has not changed in the last 50 years. The only approach taken relies on incrementally increasing the surface available for the cells to grow in the lowest volume of culture media. In other words, to accommodate as many cells as possible using the smallest volume of media. Thus, when scaling-up the manufacture of adherent cells at industrial scale, the bioreactors footprint still represents a hurdle due to the difficulties in operating and monitoring high-volumes tanks. Such approaches led to the use of cells-bead surfaces that are stirred in suspension (as we mentioned above via using microcarriers and packed bed reactors). However, the challenges associated with cell-detachment and the subsequent harvest, are still not resolved.

Moreover, forecasts on the future demand of adherent cells to be manufactured seem to go far beyond the current capabilities of established platforms. In particular, the demand for adherent cells for industries such as allogeneic cell therapies and cultured meat will increase exponentially over a relatively short period of time; meaning there is a pressing need for new and innovative enabling technologies.

**Figure 7:**
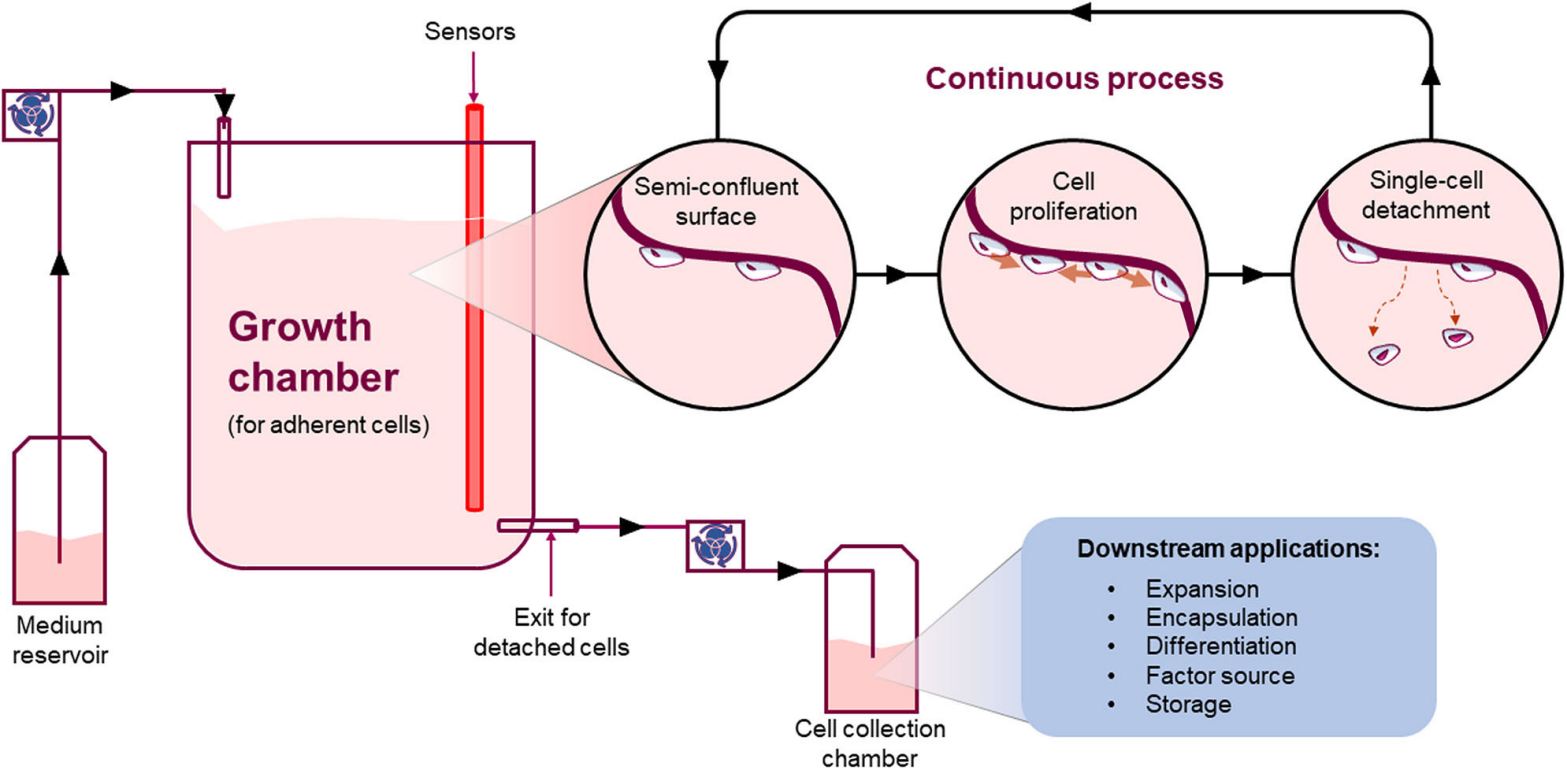
*Continuous process*. Schematic showing an example of how a continuous process for the manufacture of adherent cells could work. Such system will allow adherent cells to grow onto a surface and detach continuously as single-cells at a steady-state. The detaching cells will leave an empty space for neighboring cells to grow into maintaining a stable number of cells in culture whilst continuously harvesting single cells from the system.

An alternative approach is to develop a new bioreactor that allows the continuous manufacture of adherent cells, based on the well-known benefits of continuous bioprocessing compared to batches (Figure 7) (77). In order to do that, the critical steps are: (i) how to detach single-cells, (ii) how to maintain the system in equilibrium between detachment and proliferation (steady-state), and (iii) how to collect cells continuously.

Importantly, a continuous system has the potential to provide several advantages compared to current batch systems such as: reduction of footprint and resources, overall lower production costs, increase product quality, reproducibility and yield over time implementing a closed and automated system. For instance, a recent paper showed proof-of-concept data suggesting that an area of just 155 cm^2^ (like a medium size tissue culture flask) can generate over 1 million of cells every 24 h (76). Thus, such a small area could generate ~100 million cells when working continuously for 3 months. Additionally, a continuous system applied to adherent cell manufacture could facilitate true single-cell real time QA. In such a system, single cells are continuously detaching and moving under-flow from the area where they were growing to the next downstream process. A checkpoint could be inserted allowing a decision to progress the cell to the next downstream process or be discarded accordingly. This could be applied to each and every cell manufactured. In turn, this new way of manufacturing cells can either change the following downstream processes or drive the development of a collection system can fit with current downstream processes. Either way, it will be a critical step that has to be thoroughly considered and planned before being implemented.

In general, the benefits of continuous systems over batches are well-known and proven by different industries, and there is no reason, at this time, why they could not be applied for this specific application. However, like any new technology or process, there will be challenges and learnings but there is always the opportunity to advance and improve.

## Computational Fluid Dynamics Modeling for Scaling-Up Cell Production

Computer models have been employed to run algorithms and equations to predict the behavior of, or the outcome of many natural systems. Numerical simulations have become a resourceful tool not only for predictions, but also to accelerate the development of systems and devices in both natural and human systems (80). In particular, computational fluid dynamics (CFD) is a branch of fluid mechanics that uses numerical analysis and data structures to analyze and solve problems that involve fluid flows. Computers are used to perform the calculations required to simulate the free-stream flow of the fluid, and the interaction of the fluid (liquids and gases) with surfaces defined by boundary conditions.

Since design, construction, and evaluation of bioreactors for large-scale production is costly and time consuming, computational methods may give some insights into the fluid mechanics within bioreactors. Thus, critical limiting factors, such as insufficient mixing as well as inhomogeneous nutrient and oxygen mass transfer, may be identified early in the process design (81). CFD analysis can provide details of fluid velocities, pressures, solute or particle concentrations, temperatures, stresses, and heat/mass fluxes throughout the flow domain (67, 82). These are all important parameters to design bioreactors and scaling-up strategies. Specifically for bioreactor design, CFD is a resourceful tool to address important questions and investigate optimal parameters such as reactor type and dimensions, gas spargers design, foaming/foam control, hydrodynamic stability, mass transfer capacity, mixing, dissolved oxygen concentration/distribution as well as controversial topics such as bubble-induced cell damage (15, 67). In addition, process critical fluid flow parameters, which are hard or even impossible to measure, can be predicted by CFD (83). For instance, in the case of shear sensitive cells, the power input has to be found optimal to generate sufficient mass transfer without causing critical shear stress levels that can ultimately damage the cells (81).

CFD simulations of bubble columns, air-lift reactors or stirred tanks have been part of the work routine among chemical engineers (67). CFD analysis has been used to predict the flow behavior inside capillaries in ultra-filtration devices (84), which can be applicable for cell separation and/or cell concentration purposes (67). CFD has been also used as tool to help to understand bubbles coalescence (bubble burst), caused by gases mass transfer in the bioreactor environment, which has been a controversial subject in bioprocessing for decades (81, 83).

CFD has been widely performed for stirred-tank bioreactors at various volume scales (85, 86). Besides the classical bioreactors made of glass or stainless steel, the fluid flows in small (87), bench top (88), and pilot scale (89), helping to identify, for example, death zones or stagnant zones, where fluid flows very slowly or does not flow at all (90), impacting in the mass transfer and in the final product viability. Li et al. explored the CFD model to estimate the mass transfer and mixing performance of a reactor to scale-up cell production for cultured meat applications (91). The same approach is already widely used for the design and manufacture of several medical devices and is well-suited for conducting optimization studies to evaluate far more design alternatives than the build and test method, impacting in the reduction of design cycle time (15, 67).

We could say that CFD is a “weather forecast” for bioprocessing engineers assisting them to predict *a priori* the behavior of adherent cells growing within bioreactors. Successful scale-up of bioprocesses requires that laboratory-scale performance is equally achieved during large-scale production to meet economic constraints (92). Most importantly, CFD can reduce time- and cost-intensive trial-and-error experiments, which is especially important if the availability of the biological material is limited (i.e., primary tissues or stem cells). When the main engineering parameters, such as power input, mixing time, and (oxygen) mass transfer coefficient, are simulated and predicted, it is possible to optimize cell growth and productivity, whilst maintaining high product quality (81).

## Concluding Remarks

There are a considerable number of technologies available to scale-up cell manufacture. These have been developed mainly to be used by the biotech and pharma industries. At the moment, there are no commercially available bioreactors that are designed *ad hoc* for cultured meat applications. Thus, it is likely that two different strategies are adopted by groups working in the cultured meat field: (1) try to adapt their cell manufacture process around existing batch technologies; (2) develop manufacturing platforms in house, that are very specific for their needs. It has to be considered that this field will have to generate cell numbers that are possibly the highest among all existing industries (1). However, the question remains as to whether current technologies will be capable of meeting such considerable cell demand. We believe, based upon current commercially available technologies, that batch processes will not be capable of generating the required number of adherent cells in an efficient way. Moreover, we believe that a drastic change in the way we have been growing and manufacturing these types of cells must happen, developing new systems bringing, for instance, the well-known advantages of continuous bioprocessing into play.

Great ideas and honorable goals in this field need to be coupled with new and highly innovative enabling technologies to support them. The great challenge of efficiently producing cultured meat products at scale, gives the possibility to develop new concepts and bioprocesses that did not exist before, driving innovation across multiple disciplines along with it. We believe that continuous cell manufacture could be one of these new concepts helping cultured meat companies achieving their goal. But surely, more are yet to come to drive innovation even further.

## Author Contributions

CB, JA, and LD wrote the manuscript. MM and LG edited and provided intellectual contribution to the manuscript. CC edited, provided intellectual contribution, and approved for publication. All authors contributed to the article and approved the submitted version.

## Conflict of Interest

JA, LD, MM, and LG are employed by CellulaREvolution Ltd. CellulaREvolution Ltd is a spin-out from Newcastle University developing a novel continuous bioreactor to manufacture adherent cells. MM, LG, and CC are co-founders and shareholders of CellulaREvolution Ltd. The remaining author declares that the research was conducted in the absence of any commercial or financial relationships that could be construed as a potential conflict of interest.
